# Treatment patterns and clinical outcomes in 157 patients with extensive-stage small cell lung cancer: real-world evidence from a single-center retrospective study

**DOI:** 10.3389/fonc.2023.1287628

**Published:** 2023-12-04

**Authors:** Yumin Zheng, Kexin Tan, Aolin Wang, Xingyu Lu, Huijing Dong, Jia Li, Huijuan Cui

**Affiliations:** ^1^ Graduate School, Beijing University of Chinese Medicine, Beijing, China; ^2^ Department of Integrative Oncology, China-Japan Friendship Hospital, Beijing, China

**Keywords:** extensive-stage small-cell lung cancer, treatment pattern, retrospective study, immunotherapy, chemotherapy

## Abstract

**Background:**

Immune checkpoint inhibitors (ICIs) have changed the therapeutic options for extensive-stage small-cell lung cancer (ES-SCLC). In this real-world study, we analyzed the treatment patterns in patients with ES-SCLC and evaluated the efficacy of chemotherapy combined with immunotherapy as first-line therapy.

**Methods:**

A retrospective analysis was performed on patients with ES-SCLC who received treatment at China-Japan Friendship Hospital (Beijing, China) between August 1, 2020, and April 30, 2023. The treatment patterns appeared in the form of Sunburst Chart and Sankey diagram. The survival analyses were conducted by Kaplan-Meier curves.

**Results:**

A total of 157 patients with ES-SCLC were retrospectively included. According to first-line therapy, patients were divided into the chemotherapy (CT) group (n=82) and chemo-immunotherapy (CIT) group (n=75). The median treatment lines were 2[1, 2] and cycles were 8[5, 12], respectively. 82 patients received the second line of therapy, followed by 37 for the third, 15 for the fourth, 11 for the fifth, and 5 for the sixth. Overall, the treatment patterns involved 11 options including 12 chemotherapy regimens, 11 ICIs, and 4 targeted agents. The second-line treatment pattern had the most options (9) and regimens (43). In the first 3 lines, chemotherapy was the largest proportion of treatment options. The addition of ICIs prolonged progression-free survival from 6.77 (95% confidence interval [CI], 6.00-7.87) to 7.33 (95% CI, 6.03-9.80) months (hazard ratio [HR]=0.67, 95% CI, 0.47-0.95; P=0.025), overall survival from 12.97 (10.90-23.3) to 14.33 (12.67-NA) months without statistically significant difference (HR=0.86, 95% CI, 0.55-1.34; P=0.505).

**Conclusion:**

The treatment options of patients with ES-SCLC are more diversified. Combination therapy is the current trend, where chemotherapy is the cornerstone. Meanwhile, ICIs participate in almost all lines of treatment. However, the clinical efficacy remains barely satisfactory. We are urgently expecting more breakthrough therapies except immunology will be applied in the clinic.

## 1 Introduction

Small-cell lung cancer (SCLC) comprises approximately 13-17% of all lung cancer (1), and the 5-year survival rate is approximately 6.4% (2). According to the staging criteria of the American Veterans Lung Cancer Association (VALG), patients with SCLC are classified as limited disease (LD) and extensive stage (ES). Nearly 65% of patients are ES-SCLC when they were diagnosed, and the 2-year survival rate with chemotherapy is less than 10% (3). In short, ES-SCLC is a refractory cancer with low survival and a high recurrence rate. Platinum-based chemotherapy is the mainstay treatment for ES-SCLC, and the treatment options are relatively limited with slow development.

With the development of precision medicine, the clinical stagnation of SCLC for more than 20 years has begun to break. Immune checkpoint inhibitors (ICIs), targeted antiangiogenic drugs, and other novel chemotherapeutic agents have changed the treatment patterns for patients with ES-SCLC. ICIs combined with chemotherapy have been approved as first-line treatment, prolonging overall survival (OS) for about 2-2.5 months (4–6). For patients with relapsed SCLC, anlotinib as third- or further-line therapy significantly improved median progression-free survival (PFS) (4.0m) and OS (7.3m) (7). However, clinical trials testing new drugs and new drug combinations failed to further change the standard of care. With regards of first-line treatment, the phase III SKYSCRAPER-02 testing tiragolumab, a novel anti-TIGIT inhibitory immune checkpoint agent, combined with atezolizumab and chemotherapy did not prolong patients’ survival rates. On the other hand, for pre-treated patients, trials involving drugs such as lurbinectedin (8), olaparib (9) and rovalpituzumab tesirine (10), all failed to reach their primary endpoints.

Currently, patients diagnosed with ES-SCLC are experiencing a change of treatment sequence with the development of immune therapies. ICIs have dramatically revolutionized the selection of first-line treatment, from the initial to maintenance phase. They also have a significant impact on subsequent treatment options given their superior survival benefits. There are numerous therapeutic regimens and drug combinations used in clinical practice, which may be influenced by various real-world factors. As a result, the treatment sequence has become more diversified, gradually deviating from the classical scheme. Thus, it is essential to summarize the change in treatment patterns for patients with ES-SCLC. It will assist clinicians in developing comprehensive treatment plans.

Therefore, this study primarily delineates the treatment patterns of patients diagnosed with ES-SCLC including therapeutic regimens. Furthermore, based on real-world data, the efficacy of chemotherapy combined with immunotherapy as the first-line therapy is also reported.

## 2 Materials and methods

### 2.1 Patients

The retrospective study enrolled patients with ES-SCLC who received treatment at China-Japan Friendship Hospital (Beijing, China) from August 1, 2020 to April 30, 2023. The last follow-up was conducted on June 1, 2023. The present retrospective study was performed by reviewing the medical records of patients under the approval of the Institutional Ethics Review Committee of the China-Japan Friendship Hospital (2023-KY-134). The study protocol was in accordance with the Declaration of Helsinki. The requirement for written informed consent was exempt owing to the retrospective nature of this study.

### 2.2 Inclusion and exclusion criteria

Patients eligible for inclusion met all the following criteria: (1) The patient was pathologically diagnosed with SCLC, and the disease was divided into ES-SCLC according to the VALG staging system. (2) Clinical medical record of the patient was available and complete. (3) The patient received at least 2 cycles of therapy.

Exclusion criteria were as follows: (1) The histological pathology combined with other cellular components, such as adenocarcinoma or large cell component. (2) Patient was enrolled in other clinical trials, and the investigational drug was unclear. (3) The patient combined with other malignancies which was on active treatment. (4) The patient received ablation or surgical resection of tumor lesions before first-line systemic treatment.

### 2.3 Data collection

The data for each patient was extracted from the medical records including basic information, clinical characteristics, treatment patterns, and safety data. The basic information included hospitalization number, age, gender, body mass index (BMI), smoking, alcohol consumption, family history, medical history, and the Eastern Cooperative Oncology Group (ECOG) score. Clinical characteristics included diagnosis date, diagnostic modality, Ki-67 index, detailed TNM classification, lymphatic metastasis (hilar, mediastinal, supraclavicular, and other lymph node [LN]), organ metastasis (pleura, bone, lung, brain, and liver), and radiotherapy. Treatment patterns included therapeutic agents, treatment cycles, and lines. Safety data were collected during first-line therapy including adverse events (AEs) and serious AEs.

### 2.4 Outcomes and assessments

The primary endpoints were PFS and OS. PFS was defined as the duration from initial therapy to disease progression or death due to any cause. OS was defined as the time from the start of any treatment until the date of death of any cause or last day of follow-up. All patients were actively followed up until June 1, 2023. The follow-up information was obtained by telephone or directly from the electronic medical record system documents. Finally, the assessments of AEs were performed according to the National Cancer Institute Common Terminology Criteria for Adverse Events (NCI-CTCAE) Version 5.0.

### 2.5 Statistical analysis

Continuous variables were described by using mean ± standard deviation (SD) or median and interquartile range (IQR) according to the Kolmogorov-Smirnov-test. The difference between groups was compared using Student’s t-test or the Mann-Whitney U-test. Categorical variables were described by frequencies and percentages. The difference was compared using Pearson’s chi-squared and Fisher’s exact tests. PFS and OS were estimated using the Kaplan-Meier curves of survival, and differences in time distributions were compared using the log-rank test; the estimated median time (months) and 95% confidence interval (CI) were presented. Cox proportional hazard model was used to evaluate prognostic factors for PFS and OS by univariate and multivariate analysis. The factors with p-value < 0.05 at univariate Cox analysis were adopted into multivariate Cox regression to determine independent prognostic factors. All statistical analyses were performed by R 4.0.3 and SPSS 20.0 software. All p-values were two-sided, and statistical significance was set at p-value <0.05.

## 3 Results

### 3.1 Baseline characteristics

**Figure 1:**
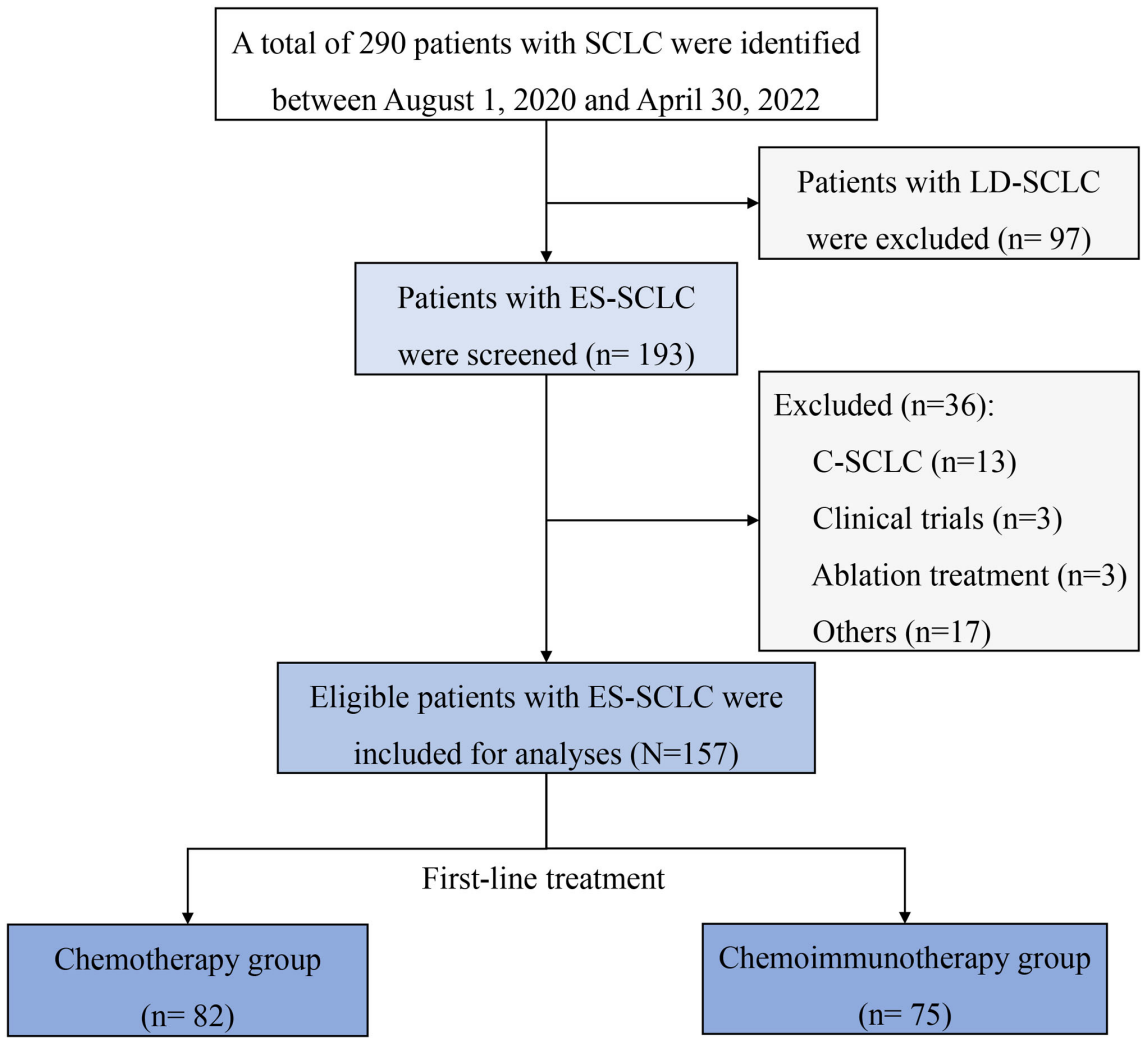
Diagram of patient’s selection process. C-SCLC, combined small-cell lung cancer; ES-SCLC, extensive-stage small-cell lung cancer; LD-SCLC, limited-disease small-cell lung cancer; SCLC, small-cell lung cancer.

A total of 157 patients with ES-SCLC were retrospectively included in the China-Japan Friendship Hospital, between August 1, 2020, and April 30, 2023. The diagram of patient selection process is shown in **Figure 1**. Our population had a median age of 63 years, among which 82.8% were males and 55.41% were smokers. The most common comorbid conditions were cardiovascular diseases (47.77%). 114 (72.61%) patients were diagnosed by bronchoscopy and 55 (37.93%) patients received radiation therapy. The locations of LN metastasis were mostly mediastinum (84.71%) and pulmonary hilum (71.97%). The most common metastatic sites were pleura (51.59%) and bone (33.76%). 27 patients (17.2%) had brain metastasis (BM) at baseline. Only six patients were staged as M0.

According to first-line therapy, there were 82 (52.87%) patients in the chemotherapy (CT) group and 75 (47.77%) patients in the chemo-immunotherapy (CIT) group. The baseline characteristics were relatively balanced between the two groups without significant differences, except for smoking, alcohol consumption, T staging, and Ki-67 index. The characteristics of patients with ES-SCLC are shown in **Table 1**.

**Table 1:** Clinical characteristics of patients with ES-SCLC stratified by first-line treatment.

	Overall	CT Group	CIT Group	*P*-Value
Number, n	157	82 (52.87)	75 (47.77)	
Gender				1.000
Female	27 (17.20)	14 (17.07)	13 (17.33)	
Male	130 (82.80)	68 (82.93)	62 (82.67)	
Age (years)				
Mean (SD)	63.36 (8.84)	63.51 (8.85)	63.19 (8.88)	0.819
>65	67 (42.68)	37 (45.12)	30 (40.00)	0.627
BMI (kg/m^2^)				0.070
18.5-24	70 (44.59)	32 (39.02)	38 (50.67)	
>24	79 (50.32)	43 (52.44)	36 (48.00)	
<18.5	8 (5.10)	7 (8.54)	1 (1.33)	
Smoking	87 (55.41)	61 (74.39)	26 (34.67)	<0.001*
Alcohol consumption	72 (45.86)	28 (34.15)	44 (58.67)	0.004*
Family history	20 (12.74)	9 (10.98)	11 (14.67)	0.650
Comorbid conditions				
Pulmonary	27 (17.20)	18 (21.95)	9 (12.00)	0.150
Cardiovascular	75 (47.77)	37 (45.12)	38 (50.67)	0.593
Endocrine	48 (30.57)	21 (25.61)	27 (36.00)	0.216
Digestive	28 (17.83)	12 (14.63)	16 (21.33)	0.375
ECOG				0.148
0	79 (50.32)	36 (43.90)	43 (57.33)	
1	56 (35.67)	35 (42.68)	21 (28.00)	
2	22 (14.01)	11 (13.41)	11 (14.67)	
Diagnostic method				0.444
Bronchoscopy	114 (72.61)	57 (69.51)	57 (76.00)	
Histopuncture	40 (25.48)	24 (29.27)	16 (21.33)	
Pleural fluid tissue	3 (1.91)	1 (1.22)	2 (2.67)	
Location of LN metastasis				
Hilar LN	113 (71.97)	56 (68.29)	57 (76.00)	0.370
Mediastinal LN	133 (84.71)	71 (86.59)	62 (82.67)	0.646
Clavicular LN	53 (33.76)	26 (31.71)	27 (36.00)	0.690
Others	23 (14.65)	12 (14.63)	11 (14.67)	1.000
Type of metastases				
Pleura	81 (51.59)	41 (50.00)	40 (53.33)	0.797
Bone	53 (33.76)	23 (28.05)	30 (40.00)	0.158
Lung	38 (24.20)	24 (29.27)	14 (18.67)	0.173
Brain	27 (17.20)	11 (13.41)	16 (21.33)	0.271
Liver	22 (14.01)	10 (12.20)	12 (16.00)	0.649
T				0.007*
T1-2	58 (36.94)	39 (47.56)	19 (25.33)	
T3-4	99 (63.06)	43 (52.44)	56 (74.67)	
N				0.222
N0-1	13 (8.28)	8 (9.76)	5 (6.67)	
N2	59 (37.58)	35 (42.68)	24 (32.00)	
N3	85 (54.14)	39 (47.56)	46 (61.33)	
M				0.413
M0	6 (3.82)	2 (2.44)	4 (5.33)	
M1a	57 (36.31)	34 (41.46)	23 (30.67)	
M1b	32 (20.38)	17 (20.73)	15 (20.00)	
M1c	62 (39.49)	29 (35.37)	33 (44.00)	
Stage				0.343
III	6 (3.82)	2 (2.44)	4 (5.33)	
IVA	88 (56.05)	50 (60.98)	38 (50.67)	
IVB	63 (40.13)	30 (36.59)	33 (44.00)	
KI67 index	80 [73.75, 90]	80 [70, 90]	82.5 [80, 90]	0.021*
Radiotherapy	55 (37.93)	32 (45.07)	23 (31.08)	0.118

BMI, body mass index; CT, chemotherapy; CIT, chemo-immunotherapy; ECOG, Eastern Cooperative Oncology Group; LN, lymph nodes; T, tumor staging; N, regional lymph node staging; M, metastasis staging. *P<0.05

### 3.2 Treatment patterns

#### 3.2.1 The lines and cycles of treatment

By the last follow-up on June 1, 2023, 157 patients with ES-SCLC received first-line therapy, followed by 82, 37, 15, 11, and 5 patients who underwent second to sixth line of therapy respectively. The median treatment lines were 2[1, 2] and cycles were 8[5, 12], respectively. The median treatment cycles from the first to sixth line were 6, 3, 3, 3, 3, and 1. The CT and CIT groups had significant differences in the treatment lines (P=0.013) and treatment cycles of the first line (P=0.001). As for subsequent lines, there were no significant differences in treatment cycles between the two groups. The lines and cycles of treatment for 157 patients with ES-SCLC are shown in **Table 2**.

**Table 2:** The treatment lines and cycles were received in 157 patients with ES-SCLC.

	Overall	CT Group	CIT Group	*P*-Value
Number, n	157	82 (52.87)	75 (47.77)	
Therapy lines (median [IQR])	2 [1, 2]	2 [1, 3]	1 [1, 2]	0.013*
Total treatment cycles	8 [5, 12]	8 [6, 11.75]	8 [5, 13]	0.621
First line	6 [4, 7]	6 [4, 6]	6 [5, 8.5]	0.001*
Second line	3 [2, 5]	3 [2, 4.5]	3 [2, 5]	0.861
Third line	3 [2, 4]	3 [2, 4]	3 [2.25, 4]	0.773
Fourth line	3 [2, 4]	3.5 [2, 4.75]	2 [2, 3]	0.259
Fifth line	3 [2, 6]	5 [1.5, 8.5]	2.5 [2, 3.25]	0.566
Sixth line	1 [1, 2]	1.5 [1, 3.5]	1 [1, 1]	0.429

CT, chemotherapy; CIT, chemo-immunotherapy; *P<0.05.

#### 3.2.2 Therapeutic regimens

**Figure 2:**
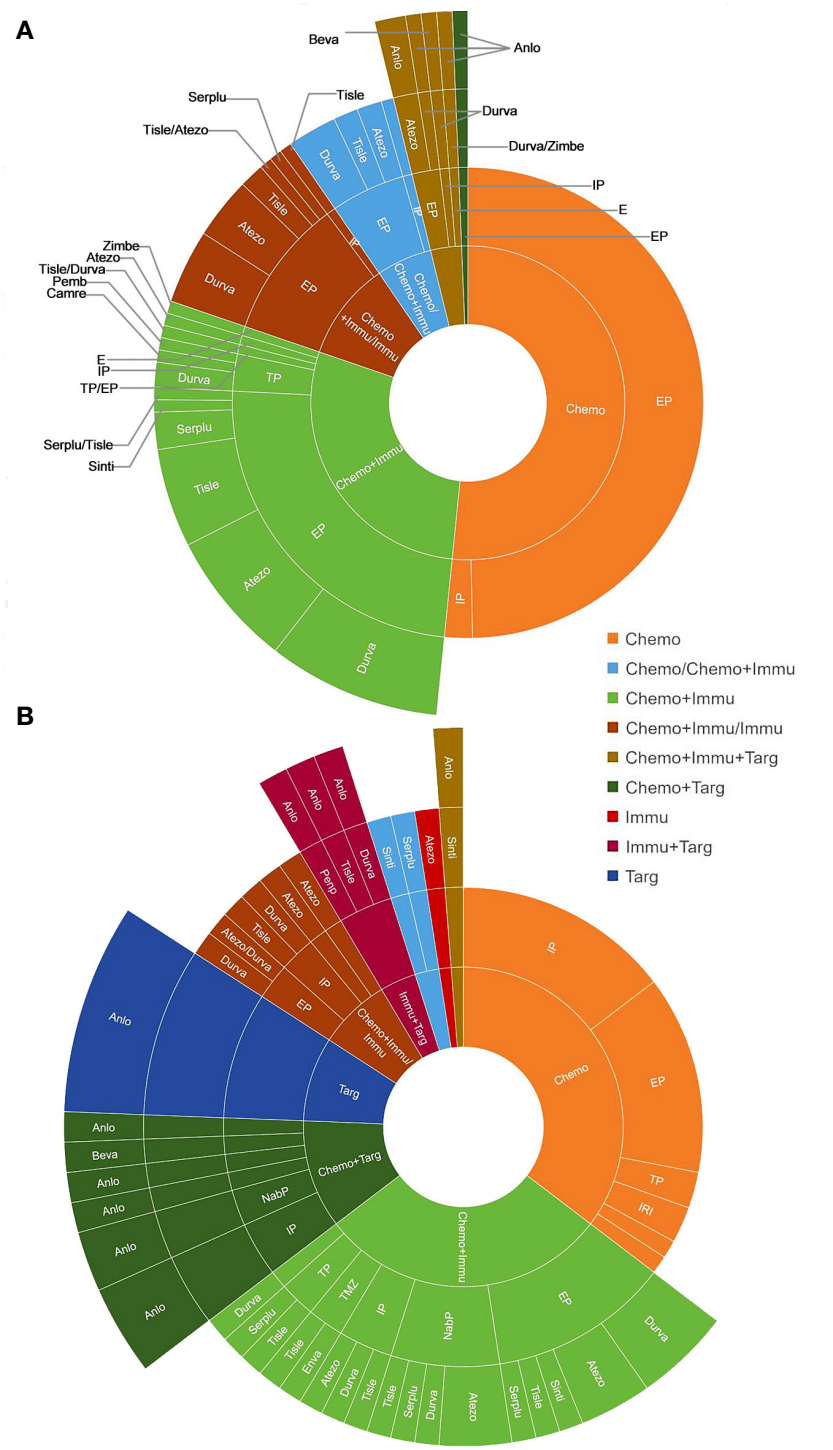
The Sunburst Chart of **(A)** first-line and **(B)** second-line therapeutic regimens.

**Figure 3:**
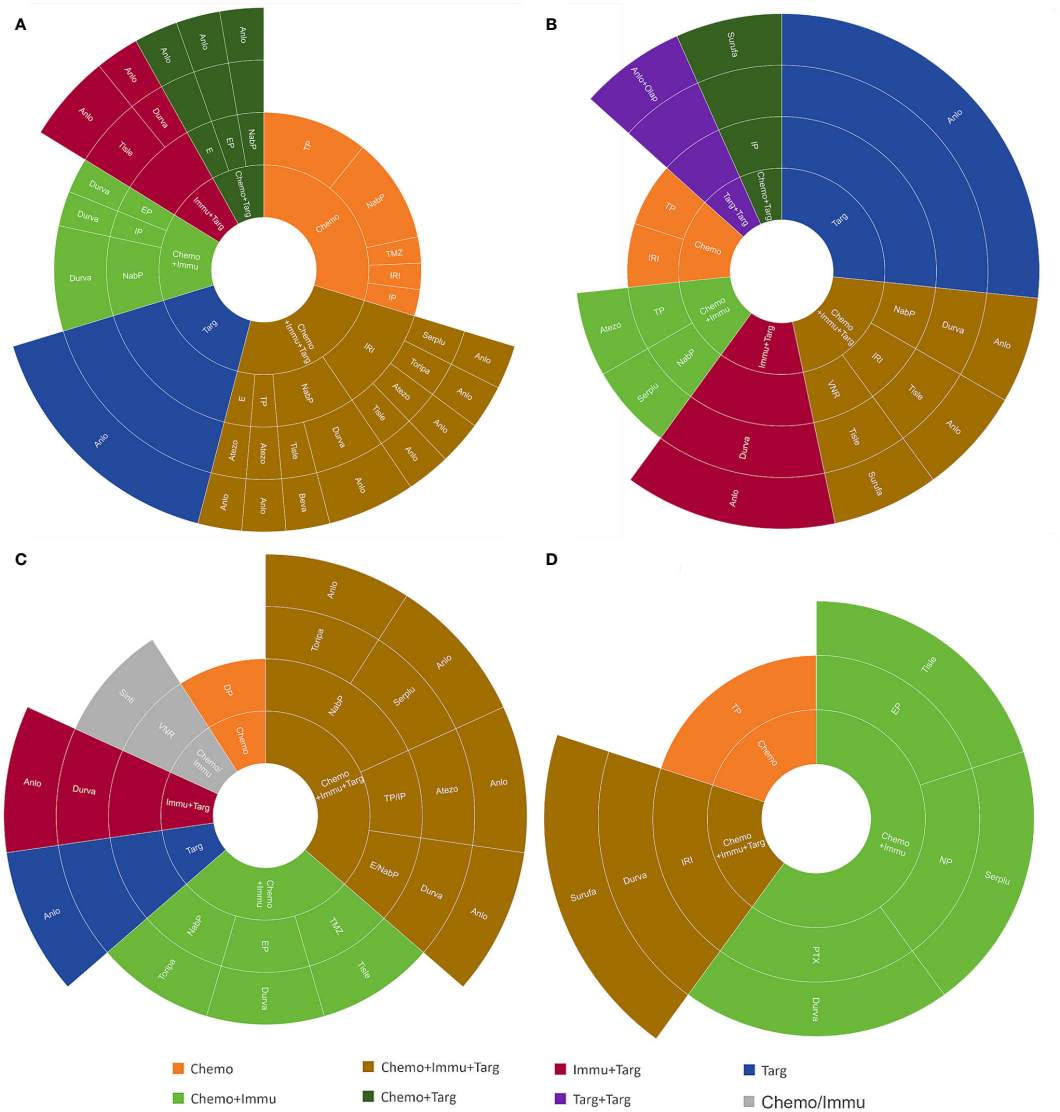
The Sunburst Chart of **(A)** third-line, **(B)** fourth-line, **(C)** fifth-line, and **(D)** sixth-line therapeutic regimen.

**Figure 4:**
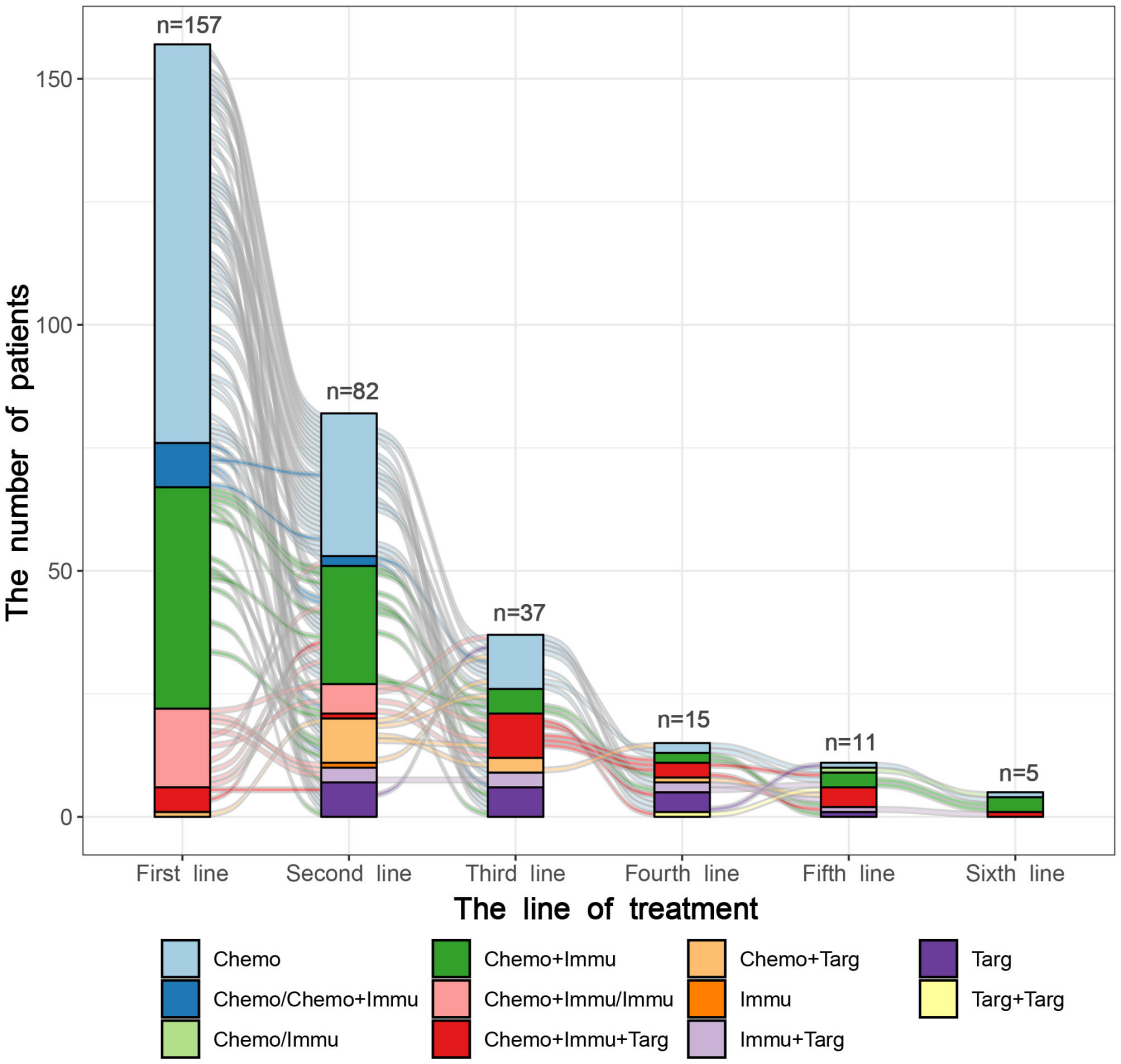
The Sankey diagram of treatment options from first to sixth line in 157 patients with SCLC.

The treatment regimens of the first six lines were visualized in **Figures 2**, **3**. by Sunburst Chart, respectively. Inner to outer circles represented treatment options, chemotherapy regimens, ICIs, and targeted agents, respectively. In total, there were 11 kinds of treatment options including chemotherapy, immunotherapy, and targeted therapy, either alone or in combination with one another therapies. In the first 3 lines, the largest proportion of treatment options was chemotherapy. The main therapeutic options from lines 4 to 6 were targeted therapy, immunochemotherapy plus targeted therapy, and immunochemotherapy, respectively. The detailed treatment options and regimens were summarized in **Supplementary Table 1**. Among them, the second line had the highest number of treatment options (n=9) and regimens (n=43). To provide a more intuitive depiction of the change, a Sankey diagram is presented illustrating the treatment options across the first to sixth lines in **Figure 4**.

As for treatment regimens, 157 patients with ES-SCLC involved 12 chemotherapy regimens, 11 ICIs, and 4 targeted agents. The first-line treatment regimens had the greatest variety of ICIs (n=8), while the second line had the widest selection of chemotherapy regimens (n=9). The most common regimens for the first-line treatment included etoposide plus cisplatin (EP) (n=78), EP plus durvalumab (n=14), and EP plus atezolizumab (n=11). The most widely used regimens for the second-line treatment were irinotecan with platinum (IP) (n=12), EP (n=11), and anlotinib (n=7). Anlotinib was also the most frequently used treatment regimen in the third (n=6) and fourth (n=4) lines. However, there was only 1 person for each treatment regimen in the fifth and sixth lines.

### 3.3 Survival analysis

#### 3.3.1 Efficacy of treatment

**Figure 5:**
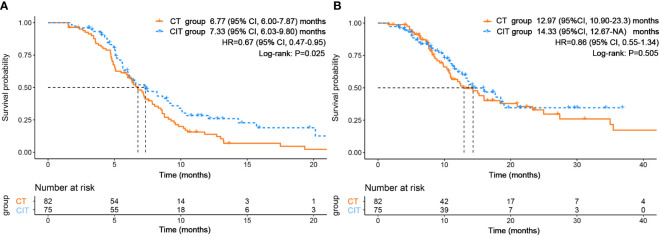
The Kaplan-Meier survival curves of **(A)** PFS and **(B)** OS between CT and CIT groups.

At the end of the follow-up period, 121 (77.1%) patients experienced disease progression or death. There were 72 (59.5%) patients in the CT group and 49 (40.5%) patients in the CIT group. In this retrospective clinical study, the median PFS of the overall study population was 7.07 months (95% CI, 6.26-8.03). The median PFS of the CIT group (7.33 months) was significantly longer compared with the CT group (6.77 months). The hazard ratio (HR) for progression or death was 0.67 (95% CI, 0.47-0.95; P=0.025) (**Figure 5A**). The median OS in the study population was 14.30 (95% CI, 12.03-18.50) months. The median OS was also longer in the CIT group compared with the CT group (14.33 vs. 12.97 months). However, the difference was not statistically significant (HR=0.86; P=0.505) (**Figure 5B**).

From the second to fourth line, PFS were 4.37 (95% CI, 3.67-5.73), 3.73 (95% CI, 3.30-5.67), and 2.20 (95% CI, 1.20-NA) months. Only in the second line did the CIT group outlive the CT group in terms of survival. The survival benefit of CIT group was not shown in third- or fourth-line therapy. The survival difference was not statistically significant between these two groups. (**Supplementary Figure 1**).

#### 3.3.2 Subgroup analysis

**Figure 6:**
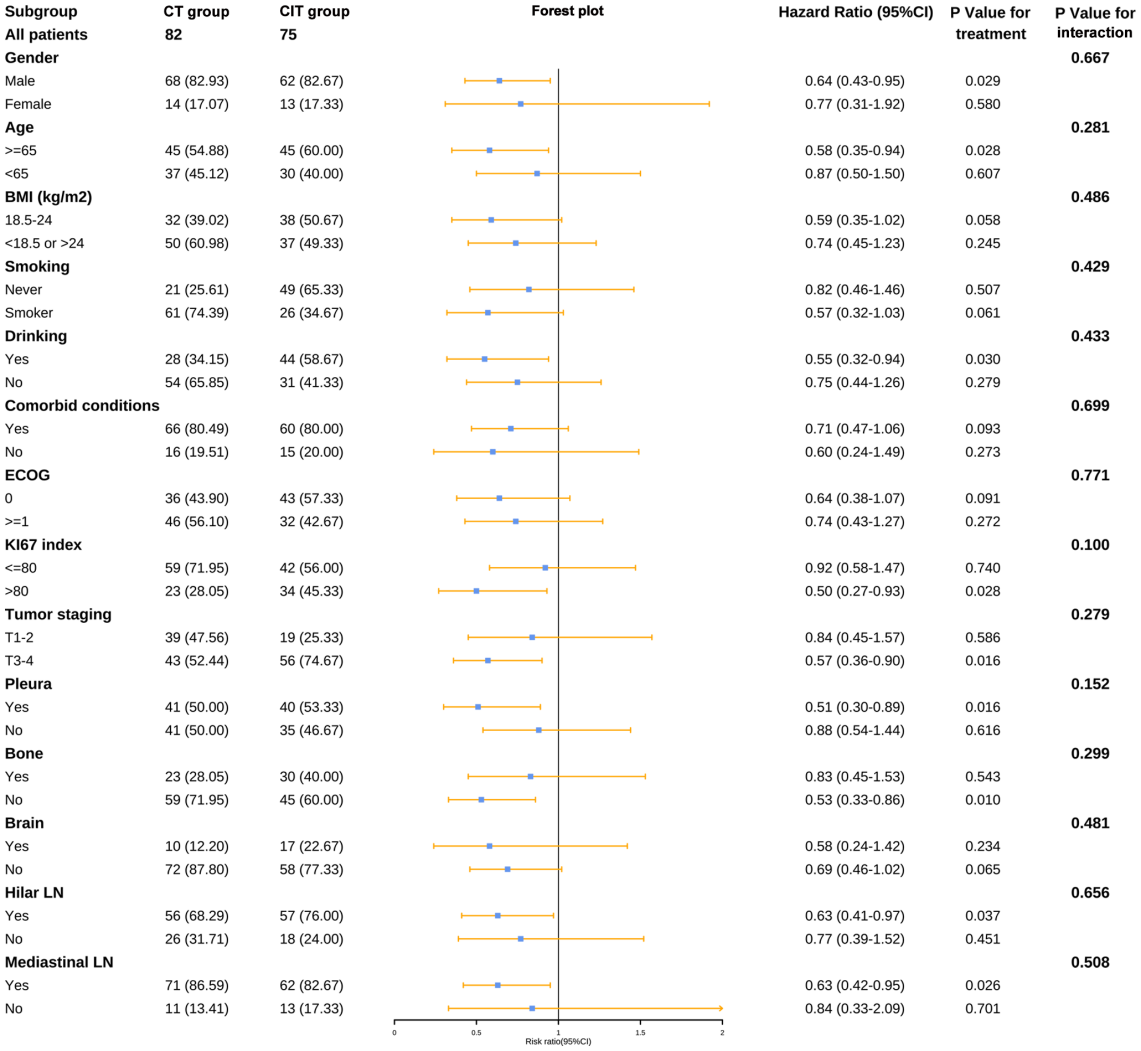
Forest plot of subgroup analysis for PFS. BMI, body mass index; CT, chemotherapy; CIT, chemo-immunotherapy; ECOG, Eastern Cooperative Oncology Group; LN, lymph nodes.

We decided to investigate if some clinical features might impact the benefit from chemotherapy plus ICIs as initial treatment. In the CIT group, individuals who were male, aged ≥65 years, drinkers, had a KI67 index greater than 80, and had pleural, bone, hilar LN, or mediastinal LN metastases exhibited a superior PFS compared to those in the CT group (**Figure 6**). As for OS, there was no subset with a statistically significant survival benefit for the CIT group (**Supplementary Figure 2**).

Given the poor prognosis of BM, our study specifically focused on patients who presented with this condition. Approximately 74.1% (20/27) of patients with BM experienced disease progression or death. PFS and OS were 6.00 (95%CI, 4.90-9.17) and 13.70 (95%CI, 9.17-NA) months, respectively. Although the differences were not statistically significant, PFS (5.50 vs. 6.00 months) and OS (10.57 vs. 18.50 months) were longer in the CIT group than in the CT one (**Supplementary Figure 3**). Furthermore, there were 63 patients with BM at the end of the follow-up in this study. The proportion of patients with BM increased from 17.2% (27/157) to 40.1% (63/157) in total. There was a 25.6% (21/82) increase in patients with BM in the CT group and 20.0% (15/75) in the CIT group.

### 3.4 Survival risk factors analysis

The potential risk factors for survival were analyzed using univariate and multivariate Cox proportional hazard models. The univariate analysis revealed that PFS was significantly associated with eight factors. In the multivariate analysis, mediastinal LN metastasis, bone metastases, liver metastases, therapy lines, and treatment cycles were identified as independent prognostic factors. Except for treatment cycles, those remaining indicators were poor prognostic factors (HR>1) for PFS (**Table 3**).

**Table 3:** Analysis of potential risk factors for PFS using univariate and multivariate Cox proportional hazard models.

	Univariable analysis		Multivariate analysis	
Characteristics	HR (95% CI)	*P*-Value	HR (95% CI)	*P*-Value
Gender (Female/Male)	1.59 (0.85-2.95)	0.144		
Age (>65/≤65)	0.86 (0.55-1.35)	0.520		
Comorbid conditions (Yes/No)	1.61 (0.87-2.98)	0.133		
Pulmonary (Yes/No)	1.02 (0.56-1.85)	0.960		
Cardiovascular (Yes/No)	1.73 (1.10-2.72)	0.017*	1.23 (0.73-2.08)	0.442
Endocrine (Yes/No)	1.61 (1.01-2.55)	0.045*	1.24 (0.75-2.05)	0.403
Digestive (Yes/No)	0.58 (0.28-1.21)	0.145		
Diagnostic method				
Histopuncture	0.91 (0.55-1.51)	0.721		
Pleural fluid tissue	5.76 (1.33-24.88)	0.019*	2.84 (0.62-12.98)	0.178
KI67 index (≤80/>80)	0.68 (0.42-1.10)	0.120		
Hilar LN (Yes/No)	1.56 (0.92-2.64)	0.100		
Mediastinal LN (Yes/No)	2.14 (1.03-4.45)	0.043*	2.90 (1.28-6.58)	0.011*
Type of metastases				
Pleura (Yes/No)	0.69 (0.44-1.09)	0.113		
Bone (Yes/No)	2.28 (1.44-3.59)	<0.001*	2.12 (1.20-3.72)	0.009*
Lung (Yes/No)	0.78 (0.46-1.35)	0.379		
Brain (Yes/No)	1.05 (0.57-1.96)	0.868		
Liver (Yes/No)	1.87 (1.04-3.36)	0.035*	1.95 (1.05-3.63)	0.036*
Therapy lines	0.80 (0.68-0.95)	0.010*	1.47 (1.09-1.99)	0.013*
Treatment cycles	0.88 (0.84-0.92)	<0.001*	0.81 (0.74-0.88)	<0.001*

CI, confidence interval; LN, lymph nodes; *P<0.05.

As to OS, it was significantly associated with pleural metastasis, therapy lines, and treatment cycles by the univariate analysis. In multivariate analysis, these three were also identified as independent prognostic factors. Except for therapy lines, pleural metastasis, and treatment cycles were good prognostic factors (HR<1) for OS (**Table 4**).

**Table 4:** Analysis of potential risk factors for OS using univariate and multivariate Cox proportional hazard models.

	Univariable analysis		Multivariate analysis	
Characteristics	HR (95% CI)	*P*-Value	HR (95% CI)	*P*-Value
Gender (Female/Male)	1.37 (0.85-2.22)	0.191		
Age (>65/≤65)	0.94 (0.65-1.34)	0.724		
Comorbid conditions (Yes/No)	1.21 (0.76-1.91)	0.421		
Pulmonary (Yes/No)	1.07 (0.66-1.74)	0.776		
Cardiovascular (Yes/No)	1.06 (0.74-1.52)	0.760		
Endocrine (Yes/No)	1.39 (0.96-2.02)	0.083		
Digestive (Yes/No)	1 (0.62-1.62)	0.986		
Diagnostic method				
Histopuncture	1.23 (0.83-1.82)	0.308		
Pleural fluid tissue	1.31 (0.32-5.38)	0.703		
KI67 index (≤80/>80)	0.69 (0.47-1.02)	0.060		
Hilar LN (Yes/No)	1.11 (0.75-1.65)	0.593		
Mediastinal LN (Yes/No)	0.81 (0.50-1.31)	0.386		
Type of metastases				
Pleura (Yes/No)	0.62 (0.43-0.89)	0.009*	0.67 (0.46-0.96)	0.030*
Bone (Yes/No)	1.34 (0.92-1.95)	0.131		
Lung (Yes/No)	1.16 (0.77-1.76)	0.479		
Brain (Yes/No)	1.22 (0.75-1.97)	0.420		
Liver (Yes/No)	1.2 (0.71-2.01)	0.496		
Therapy lines	1.18 (1.06-1.32)	0.003*	1.64 (1.35-2.01)	<0.001*
Treatment cycles	0.97 (0.95-1.00)	0.019*	0.91 (0.87-0.95)	<0.001*

CI, confidence interval; LN, lymph nodes; *P<0.05.

### 3.5 Safety

The AEs were collected to evaluate the safety of first-line treatment options. Overall, at least one AE was experienced by 91.5% (75/82) of patients in the CT group and by 93.3% (70/75) in the CIT group (**Table 5**). The occurrence of AEs was similar in the two groups. The most common AEs in both groups were anemia, nausea, and white blood cell decreased (grade 1-2). While neutrophil count decreased was the most common grade 3 or higher AE. As for immune-related adverse events (irAEs), approximately 18.7% (14/75) of the patients experienced at least one event. The most common irAEs were rash, hypothyroidism, and pneumonia. Grade 3 or higher irAEs occurred in 3 patients (4.0%), including hepatitis, myositis, and pneumonia. No treatment-related deaths were observed.

**Table 5:** Adverse events in the CT and CIT group during the first-line treatment.

Variable	CT Group (N=82)		CIT Group (N=75)	
	G1-2	G3-4	G1-2	G3-4
Any event	75 (91.5%)	23 (28.0%)	70 (93.3%)	24 (32.0%)
Abdominal distension	2 (2.4%)	0	3 (4.0%)	0
Alopecia	6 (7.3%)	0	3 (4.0%)	0
Anemia	56 (68.3%)	3 (3.7%)	38 (50.7%)	4 (5.3%)
AST/ALT increased	5 (6.1%)	0	8 (10.7%)	0
Asthenia	21 (25.6%)	0	16 (21.3%)	0
Conjunctivitis	0	0	1 (1.3%)	0
Constipation	1 (1.2%)	0	9 (12.0%)	0
Diarrhea	2 (2.4%)	0	7 (9.3%)	0
Dysphagia	5 (6.1%)	0	5 (6.7%)	0
Febrile neutropenia	0	9 (11.0%)	0	9 (12.0%)
Infusion related reaction	2 (2.4%)	0	5 (6.7%)	0
Lymphocyte count increased	24 (29.3%)	3 (3.7%)	18 (24.0%)	3 (4.0%)
Nausea	39 (47.6%)	0	36 (48.0%)	0
Neutrophil count decreased	35 (42.7%)	21 (25.6%)	26 (34.7%)	19 (25.3%)
Platelet count decreased	13 (15.9%)	5 (6.1%)	8 (10.7%)	6 (8.0%)
Serum amylase increased	2 (2.4%)	0	3 (4.0%)	0
Thromboembolic event	4 (4.9%)	0	5 (6.7%)	0
Dizziness	2 (2.4%)	0	7 (9.3%)	0
Vomiting	10 (12.2%)	0	8 (10.7%)	0
White blood cell decreased	37 (45.1%)	9 (11.0%)	31 (41.3%)	5 (6.7%)
Immune-related adverse event			14 (18.7%)	3 (4.0%)
Enteritis	–	–	1 (1.3%)	0
Hepatitis	–	–	2 (2.7%)	1 (1.3%)
Hyperthyroidism	–	–	4 (5.3%)	0
Hypophysitis	–	–	1 (1.3%)	0
Hypothyroidism	–	–	6 (8.0%)	0
Interstitial lung disease	–	–	2 (2.7%)	0
Myocarditis	–	–	1 (1.3%)	0
Myositis	–	–	2 (2.7%)	1 (1.3%)
Pneumonia	–	–	5 (6.7%)	1 (1.3%)
Pruritus	–	–	3 (4.0%)	0
Rash	–	–	8 (10.7%)	0

ALT, Alanine aminotransferase; AST, Aspartate transaminase; CT, chemotherapy; CIT, chemo-immunotherapy; G, grade.

## 4 Discussion

In clinical practice, therapeutic regimens for patients with SCLC are limited. The standard first-line treatment for ES-SCLC was platinum-based chemotherapy until immunochemotherapy was approved for clinical use (11). To the best of our knowledge, the present study is the first retrospective study summarizing treatment patterns of ES-SCLC during a period of change, encompassing therapeutic options, regimens, agents, lines, and cycles. A total of 157 patients with ES-SCLC were retrospectively included in this study. The patients were mostly male and smokers. The most common site of metastasis was bone, which was consistent with previous studies (12–14). As the first-line treatment option, the ratio of patients who received chemotherapy to chemoimmunotherapy was almost 1:1.

The treatment patterns of SCLC had been summarized during the chemotherapy-based era. Valette et al. (15) provided many aspects of treatment strategy highlighting the role of radiotherapy, subsequent lines of therapy, and the outcomes of ES-SCLC patients. The characteristics, treatment patterns, and clinical outcomes of patients with SCLC in European and Chinese populations had also been studied separately (16, 17). However, treatment regimens of subsequent lines were relatively neglected, compared to outcomes and initial treatment. The pairing of drugs was also not explicitly stated. In this study, we visualized the treatment regimens of 157 ES-SCLC from the first to sixth lines in the form of the Sunburst Chart and Sankey diagram. With regards of treatment patterns, combination therapy, such as chemoimmunotherapy, targeted therapy plus immunotherapy, and chemotherapy plus targeted therapy, were the mainstream treatments for ES-SCLC, especially in the subsequent lines. In terms of treatment regimens, ICIs were involved in all lines of treatment for patients with ES-SCLC. There were a variety of ICIs, representing durvalumab and atezolizumab. However, chemotherapy was still the main treatment choice. Regarding treatment lines and cycles, the CT group exhibited a greater number of treatment lines, whereas the CIT group demonstrated a greater number of treatment cycles. It indicates that utilizing chemoimmunotherapy as the initial treatment prefers to reduce the likelihood of resistance or disease progression. The patients diagnosed with ES-SCLC are eligible to receive multiple cycles of ICIs as maintenance therapy after completing six cycles of chemoimmunotherapy.

Currently, it is widely acknowledged that incorporating ICIs into chemotherapy as first-line treatment for ES-SCLC patients is associated with improved OS (18). Nevertheless, the conclusion was still not enough powerful. Some studies reached contradictory findings about it (19–21). In this study, there was also no statistically significant difference in survival time between the CT and CIT groups, except for PFS. Fujimoto et al. (22) thought that trial-ineligible patients may be the reason caused the difference between the clinical trial and reality.

Regarding subgroup and prognostic analyses, several results were relatively interesting. Firstly, elderly patients were more likely to benefit from chemoimmunotherapy by subgroup analysis. The finding was generally consistent with IMpower133 (23) and other studies (24, 25). Regrettably, this study failed to yield evidence supporting the therapeutic efficacy of chemoimmunotherapy in patients with BM. Nonetheless, a recent investigation involving 85 ES-SCLC patients with baseline BM demonstrated that anti programmed death ligand 1 (PD-L1) therapy could significantly prolong OS (26). The discrepancy may be attributed to the restricted number of patients with baseline BM included in our study. Secondly, the treatment line was a poor prognostic factor in this study. In contrast, Longo et al. (27) concluded that 3 and 4 chemotherapy lines correlated with longer OS. We thought the addition of ICIs might cause the difference. The result also showed that the treatment cycle was a good prognostic factor, which was consistent with clinical cognition. The population who benefited from first-line therapy mostly experienced more cycles and fewer lines of therapy. Finally, there were few statistically significant indicators for OS compared with PFS, either subgroup or prognostic analyses. We thought there were two key points to consider. On the one hand, the data of OS were immature because of insufficient follow-up duration. On the other hand, the long survival benefit of chemoimmunotherapy may not be significant versus chemotherapy. However, the latest follow-up data revealed that chemoimmunotherapy led to a sustained median OS benefit in IMpower133 (4) and CASPIAN (5). In conclusion, the target population who could benefit the most from chemoimmunotherapy as first-line treatment should continue to be explored.

At present, multiple drug regimens are being investigated for the treatment of patients with ES-SCLC. Anti-angiogenic drugs combined with other therapies become a tendency in the first-line treatment (28), such as apatinib plus camrelizumab (29) and anlotinib combined with EP (30). This therapeutic approach shows preliminary efficacy and an acceptable safety profile. Meanwhile, ongoing clinical trials with novel agents primarily concentrate on subsequent lines of treatment, including aurora kinase A inhibitors, polyadenosine diphosphate-ribose polymerase inhibitors, and so on (31, 32). Combination therapy is the current trend in patients with refractory relapse SCLC, containing multiple therapeutic agents with distinct mechanisms, for example, olaparib and durvalumab (33).

Our study did not include radiotherapy in the treatment regimen, although it had been summarized in clinical characteristics. Nowadays there is a favorable perception regarding the application of combined immunotherapy and radiotherapy in ES-SCLC patients, despite the safety, efficacy, and optimal timing of combination therapy remain unclear (34). Radiotherapy could promote the efficacy of immunotherapy and alleviate immunosuppression by converting ‘cold’ tumors into ‘hot’ tumors (35). The combination therapy of ICIs and radiotherapy shows synergistic effects in patients with NSCLC. However, no conclusion was reached regarding ES-SCLC. Some studies thought ICIs plus radiotherapy could prolong survival (36, 37), but the toxicity caused by radiation plus ICIs can limit this function (38).

There were certain limitations to this study, primarily due to the inherent flaws of a retrospective study. The retrospective collection of clinical data may introduce some bias. Firstly, treatment regimens could not be included completely, such as radiotherapy. Moreover, only intrahospital information was included in this study which mostly were standardized treatments. But in real life, the patients who had shorter overall survival or underwent multiple-line therapies were more likely to enroll in clinical trials, and take agents previously used, old chemotherapeutic or other novel drugs. Therefore, the patients with ES-SCLC would experience more treatment lines and regimens than reported. This was a drawback for the summary of subsequent treatment options. Ultimately, due to the relatively insufficient follow-up time, and the immature OS data, the long-term survival benefit of the CIT group was not apparent.

## 5 Conclusion

The treatment options of patients with ES-SCLC are more diversified. Combination therapy is the current trend, where chemotherapy is the cornerstone for treatment at various stages of disease. Simultaneously, ICIs participate in almost all the treatment lines. Chemotherapy plus ICIs can prolong survival time as a first-line treatment. However, the clinical efficacy remains barely satisfactory. We are urgently expecting more breakthrough therapies in addition to immunology will be applied in the clinic.

## Data availability statement

The raw data supporting the conclusions of this article will be made available by the authors, without undue reservation.

## Ethics statement

The studies involving humans were approved by China-Japan Friendship Hospital Research Ethics Board. The studies were conducted in accordance with the local legislation and institutional requirements. Written informed consent for participation was not required from the participants or the participants’ legal guardians/next of kin in accordance with the national legislation and institutional requirements.

## Author contributions

YZ: Writing – original draft, Conceptualization, Data curation. KT: Writing – original draft, Data curation, Formal Analysis. AW: Writing – original draft, Data curation. XL: Writing – review & editing, Visualization, Software. HD: Writing – review & editing, Methodology, Software. JL: Writing – review & editing, Software. HC: Writing – review & editing, Supervision, Conceptualization.
