# Supplementary material for: Treatment patterns and clinical outcomes in 157 patients with extensive-stage small cell lung cancer: real-world evidence from a single-center retrospective study
Source: Front Oncol. 2023 Dec 4;13:1287628. doi: 10.3389/fonc.2023.1287628 (PMC10726005; doi:10.3389/fonc.2023.1287628)
Supplement: Supplementary file 1 [file DataSheet_1.docx]

Supplementary Material

**Supplementary Table 1.** The treatment options and regimens of first six lines in 157 patients with ES-SCLC.

| The line of treatment | Treatment options | Regimen, n | Patient. n |
| --- | --- | --- | --- |
| First line | Chemo | 2 | 81 |
|  | Chemo/Chemo+Immu | 4 | 9 |
|  | Chemo+Immu | 12 | 45 |
|  | Chemo+Immu/Immu | 6 | 16 |
|  | Chemo+Immu+Targ | 4 | 5 |
|  | Chemo+Targ | 1 | 1 |
| Total | 6 | 29 | 157 |
| Second line | Chemo | 6 | 29 |
|  | Chemo/Chemo+Immu | 2 | 2 |
|  | Chemo+Immu | 17 | 24 |
|  | Chemo+Immu/Immu | 6 | 6 |
|  | Chemo+Immu+Targ | 1 | 1 |
|  | Chemo+Targ | 6 | 9 |
|  | Immu+Targ | 3 | 3 |
|  | Immu | 1 | 1 |
|  | Targ | 1 | 7 |
| Total | 9 | 43 | 82 |
| Third line | Chemo | 5 | 11 |
|  | Chemo+Immu | 3 | 5 |
|  | Chemo+Immu+Targ | 9 | 9 |
|  | Chemo+Targ | 3 | 3 |
|  | Immu+Targ | 2 | 3 |
|  | Targ | 1 | 6 |
| Total | 6 | 23 | 37 |
| Fourth line | Chemo | 2 | 2 |
|  | Chemo+Immu | 2 | 2 |
|  | Chemo+Immu+Targ | 3 | 3 |
|  | Targ+Targ | 1 | 1 |
|  | Chemo+Targ | 1 | 1 |
|  | Immu+Targ | 1 | 2 |
|  | Targ | 1 | 4 |
| Total | 6 | 11 | 15 |
| Fifth line |  |  |  |
|  | Chemo | 1 | 1 |
|  | Chemo/Immu | 1 | 1 |
|  | Chemo+Immu | 3 | 3 |
|  | Chemo+Immu+Targ | 4 | 4 |
|  | Immu+Targ | 1 | 1 |
|  | Targ | 1 | 1 |
| Total | 6 | 11 | 11 |
| Sixth line | Chemo | 1 | 1 |
|  | Chemo+Immu | 3 | 3 |
|  | Chemo+Immu+Targ | 1 | 1 |
| Total | 3 | 5 | 5 |

Abbreviations: Chemo: Chemotherapy; Immu: Immunotherapy; Targ: Targeted therapy.


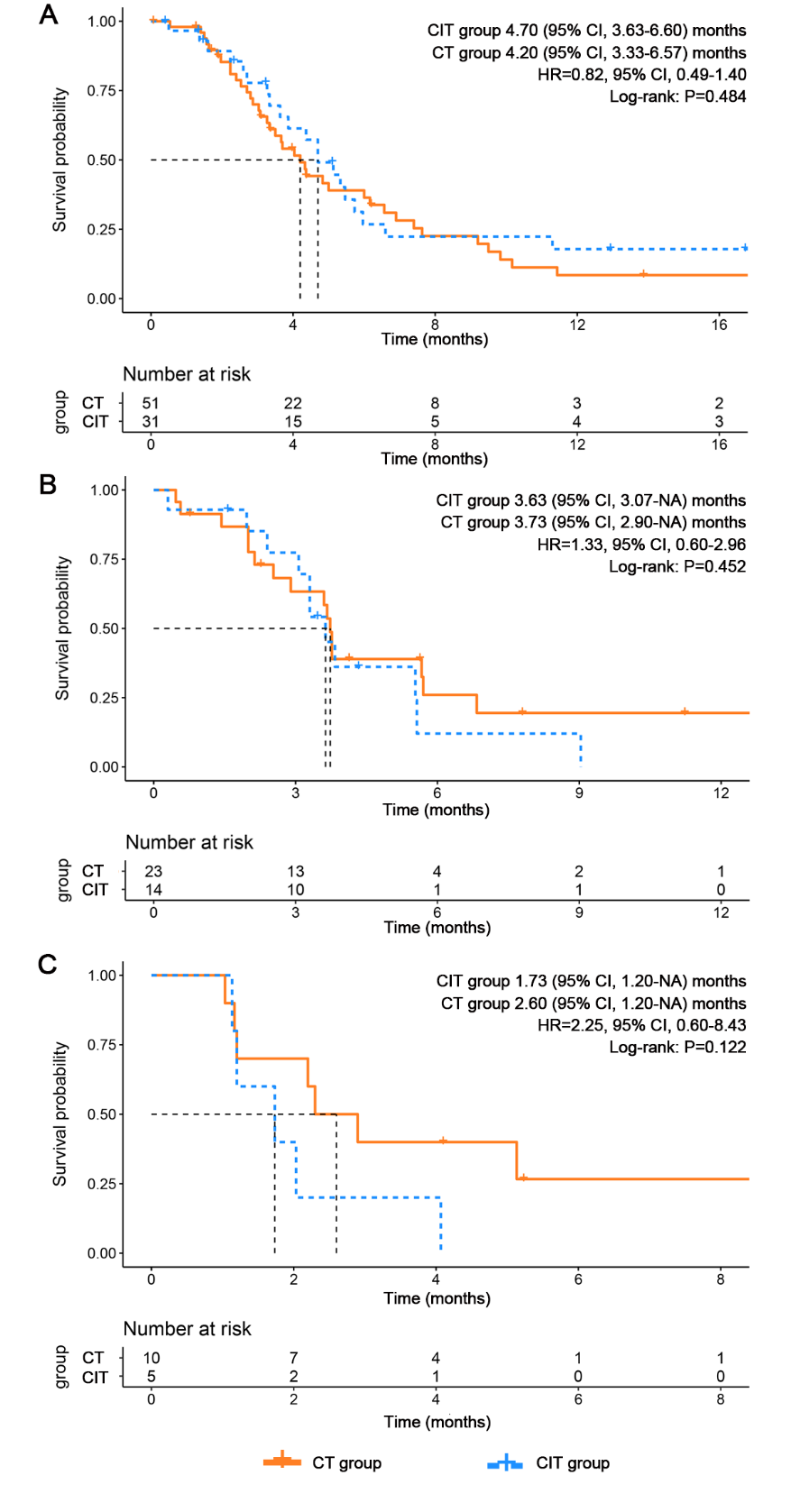


**Supplementary Figure 1.** The Kaplan-Meier survival curves of (A) the second-line PFS, (B) the third-line PFS, and (C) the fourth-line PFS between CT and CIT groups.


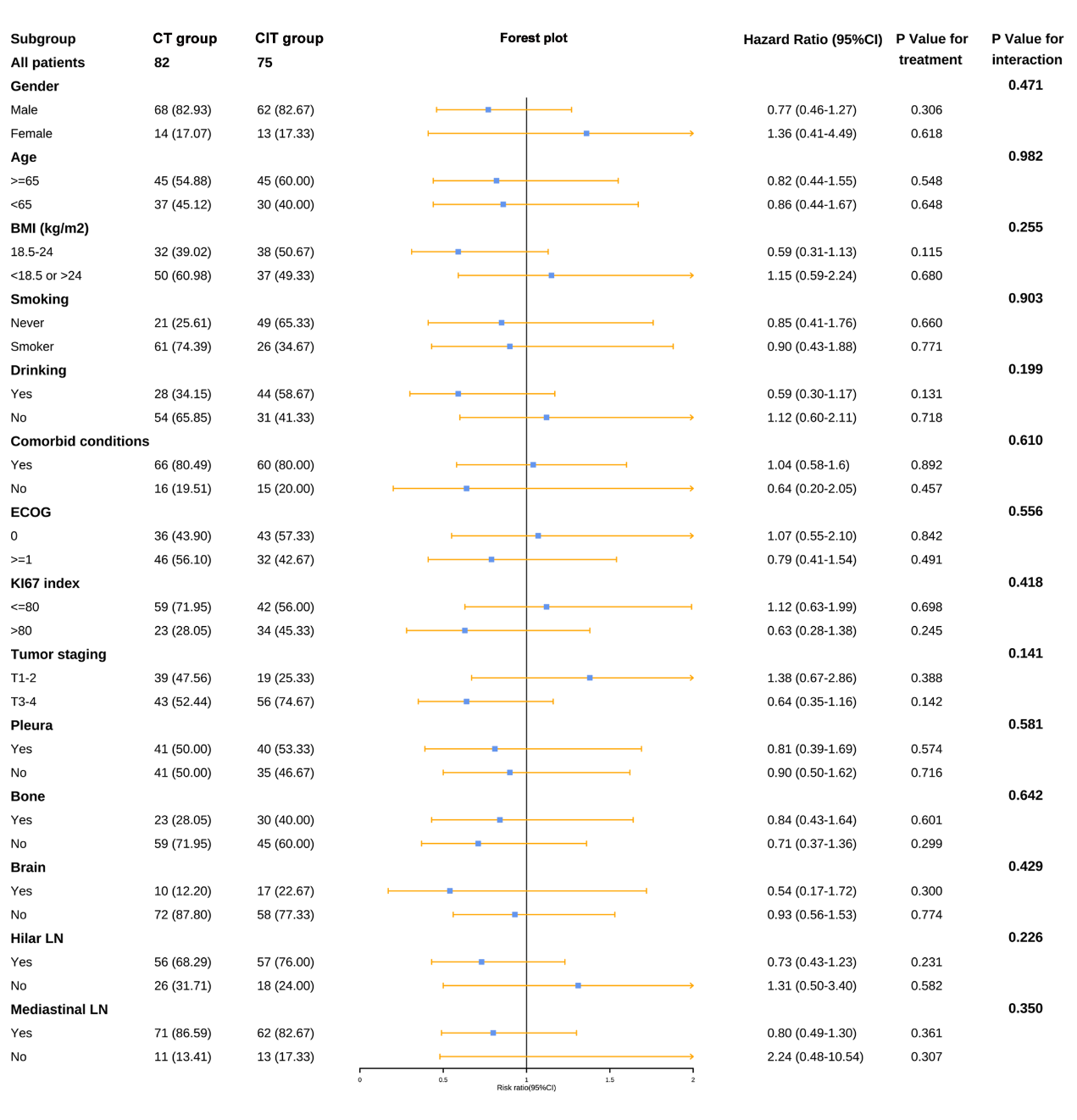


**Supplementary Figure 2.** Forest plot of subgroup analysis for OS. BMI: body mass index; CT: chemotherapy; CIT: chemo-immunotherapy; ECOG: Eastern Cooperative Oncology Group; LN: lymph nodes.


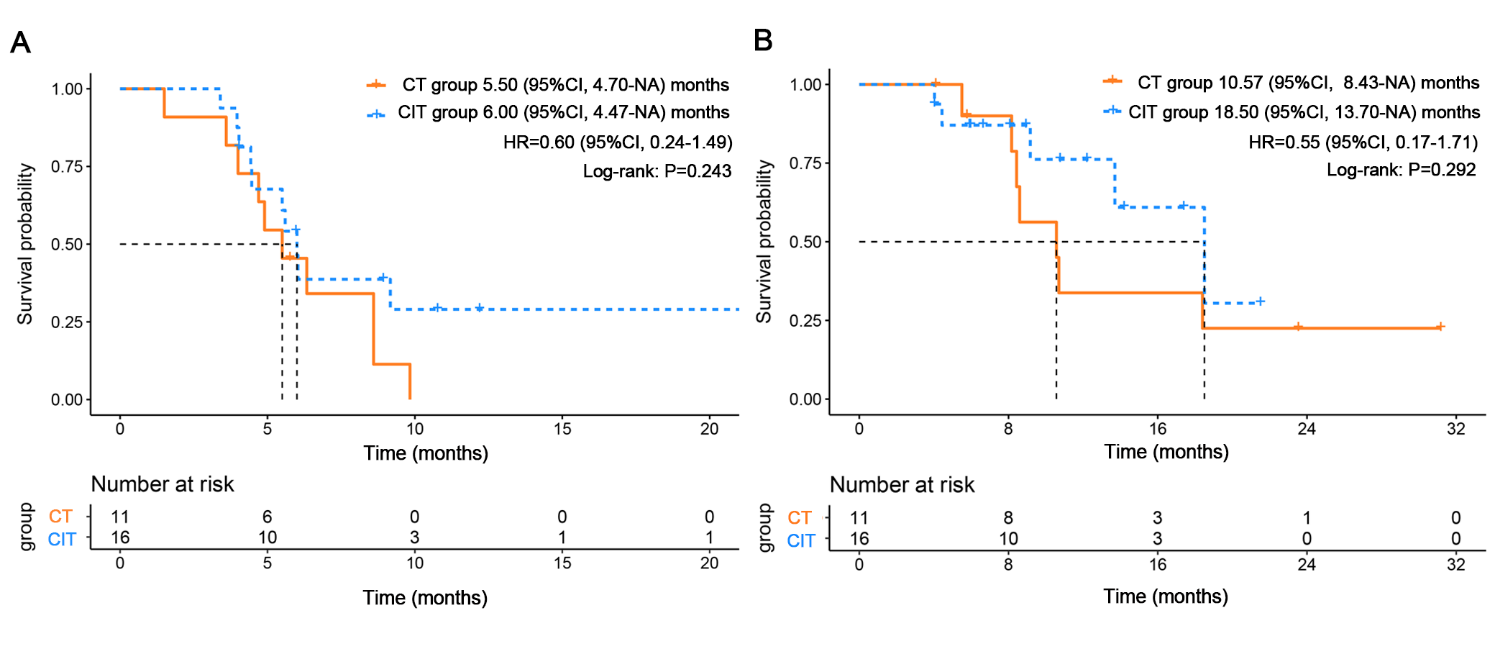


**Supplementary Figure 3.** The Kaplan-Meier survival curves of (A) PFS and (B) OS in the patients with baseline brain metastasis between CT and CIT groups.
